# Preoperative Prediction of Axillary Lymph Node Metastasis in Breast Cancer Using Mammography-Based Radiomics Method

**DOI:** 10.1038/s41598-019-40831-z

**Published:** 2019-03-14

**Authors:** Jingbo Yang, Tao Wang, Lifeng Yang, Yubo Wang, Hongmei Li, Xiaobo Zhou, Weiling Zhao, Junchan Ren, Xiaoyong Li, Jie Tian, Liyu Huang

**Affiliations:** 1 School of Life Science and Technology, Xidian University, Xi’an, Shaanxi 710071 China; 2 Department of Radiology, Shaanxi Provincial People’s Hospital, Xi’an, Shaanxi 710068 China; 3 Department of Breast Diseases, Yan’an University Affiliated Hospital, Yan’an, Shaanxi 716000 China; 4 Department of Radiology, Wake Forest School of Medicine, Medical Center Boulevard, Winston-Salem, North Carolina 27157 USA

## Abstract

It is difficult to accurately assess axillary lymph nodes metastasis and the diagnosis of axillary lymph nodes in patients with breast cancer is invasive and has low-sensitivity preoperatively. This study aims to develop a mammography-based radiomics nomogram for the preoperative prediction of ALN metastasis in patients with breast cancer. This study enrolled 147 patients with clinicopathologically confirmed breast cancer and preoperative mammography. Features were extracted from each patient’s mammography images. The least absolute shrinkage and selection operator regression method was used to select features and build a signature in the primary cohort. The performance of the signature was assessed using support vector machines. We developed a nomogram by incorporating the signature with the clinicopathologic risk factors. The nomogram performance was estimated by its calibration ability in the primary and validation cohorts. The signature was consisted of 10 selected ALN-status-related features. The AUC of the signature from the primary cohort was 0.895 (95% CI, 0.887–0.909) and 0.875 (95% CI, 0.698–0.891) for the validation cohort. The C-Index of the nomogram from the primary cohort was 0.779 (95% CI, 0.752–0.793) and 0.809 (95% CI, 0.794–0.833) for the validation cohort. Our nomogram is a reliable and non-invasive tool for preoperative prediction of ALN status and can be used to optimize current treatment strategy for breast cancer patients.

## Introduction

Breast cancer is among the most common cancer worldwide and the second cancer-related cause of death in women^1^. Axillary lymph node (ALN) status is one of the most important prognostic and diagnose factor for disease free survival and overall survival in patients with breast cancer^2^. Accurate preoperative identification of ALN status can provide clinicians with important information about their treatment decisions, such as whether axillary lymph node dissection (ALND) in surgery and postoperative adjuvant therapy are needed^2^. Currently, the intraoperative ALN status in patients with breast cancer is determined by the sentinel lymph node biopsy (SLNB)^3^. Although the accuracy of SLNB is higher, SLNB is an invasive procedure and has some complication, such as damage of blood vessels, nerve, incision infection and lymphedema^4^. Clinical investigations show that more than 50% of early-stage invasive breast cancer patients have no ALN metastasis, so any type of axillary surgeries can be considered overtreated in these cases^3^. In addition, the exact status of the dissected lymph nodes was verified by pathological examination. But reliable pathological results usually require a period of time after surgery. Therefore, there is no doubt that a non-invasive method of ALN metastasis prediction is valuable preoperative.

At present, imaging examination is used as non-invasive method to confirm the status of ALN metastasis in preoperative, such as ultrasonography, computed tomography, mammography and magnetic resonance imaging. However, imaging examination has a low diagnostic sensitivity, which may lead to a considerable proportion of ALN metastasis positive patients to be missed^5^. Lately, Dong and their colleagues have used radiomics of magnetic resonance imaging to predict sentinel lymph node metastasis in breast cancer^6^. However, magnetic resonance imaging is cost than mammography. At present, there are no study published regard to use magnetic resonance imaging or mammography to predict the status of ALN metastasis in breast cancer patients. Therefore, there is an increasing need for the development of reliable, accurate and non-invasive methods base on mammography image to predict ALN metastasis preoperatively.

Radiomics, an emerging and promising field, which is a field of medical study to convert medical images data into mineable and developable high-dimensional data via high-throughput extraction of large numbers of quantitative imaging features, and subsequently developing and analyzing these data with other patients’ characteristics to increase the power of decision support. Radiomics has been proved to be an accurate, quantitative and non-invasive method used to improve the accuracy of cancer diagnosis, prognosis and prediction^6,7^. Several recent studies have shown that radiomics has been used for the preoperative diagnosis of lymph node metastasis in some types of cancers^6–9^. Compared with magnetic resonance imaging, mammography is the most commonly used imaging examination method for the patients with breast cancer. A mammography-based radiomics tool may improve the evaluating accuracy of patients’ ALN status.

The aim of this study was to develop a mammography-based radiomics nomogram by combining radiomics signatures with clinicopathologic and immunohistochemical risk factors for the preoperative prediction of ALN metastasis in patients with breast cancer.

## Patients and Methods

### Patients

Our Institutional Review Board (Shaanxi People’s Hospital Medical Ethics Committee) approved this study and waived the need to obtain informed consent from the patients. And we confirmed that all methods were performed in accordance with the relevant guidelines and regulations. A total of 147 consecutively patients who underwent surgical treatment between January 2016 and January 2017 were included in this study. The inclusion and exclusion criteria of patients are listed in the Supplementary Data. In total, 152 patients were clinicopathologically confirmed with breast cancer, and five patients were excluded due to the indistinguishable boundary of tumor area on the mammography. Due to the 5 patients is extremely dense breast and the tumor located in the dense breast may result in indistinguishable boundary or invisible with the use of mammography^10^. Therefore, the clinical radiologists excluded the 5 patients. We randomly divided the 147 patients into primary cohort (110 patients; mean age, 55.89 ± 10.63) and validation cohort (37 patients; mean age, 50.49 ± 11.84) according to the ratio of 3:1. There are 83 ALN positive patients (61 patients in primary cohort and 22 patients in validation cohort) and 64 ALN negative patients (49 patients in primary cohort and 15 patients in validation cohort). Some studies have proved that tumor size is one of the most important factor of axillary lymph nodes metastasis in patients with breast cancer^11^. Since the research is a retrospective study, the data were all diagnosed and the tumor size of patients were most greater than 2 cm. Clinical T stage show the tumor size of patient and the total number of T2 and T3 stage is 97 (65.89%, T2: the long diameter of the cancer is greater than 2 cm and less than or equal to 5 cm; T3: the long diameter of the cancer is greater than 5 cm) in this study.

**Figure 1:**
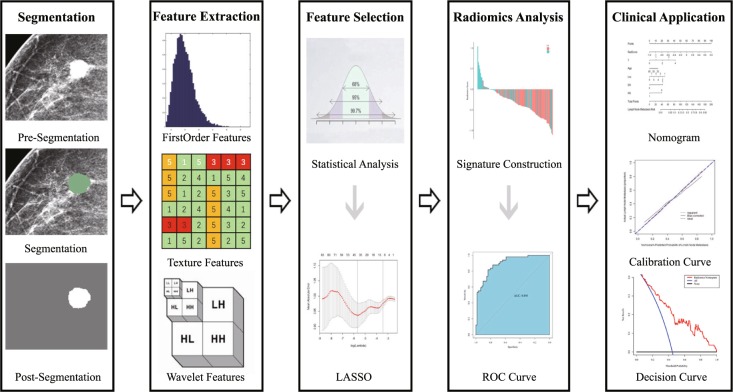
The flowchart of this study. This study includes image segmentation, feature extraction, feature selection, radiomics analysis and clinical application. The ROIs of mammography images were segmented and then 299 quantitative radiomics features extracted from post-segmentation images of individual patients. The least absolute shrinkage and selection operator (LASSO) was then used to feature selection. Thereafter, a radiomics signature was constructed and validated using the Gaussian kernel support vector machine. A radiomics nomogram was developed by incorporating the radiomics signature with clinical factors. Finally, the calibration and decision curves were used as the evaluative criteria of the radiomics nomogram. Preoperative Ultrasound-Guided Needle Biopsy of Axillary Nodes in Invasive Breast Cancer: Meta-Analysis of Its Accuracy and Utility in Staging the Axilla.

All mammography images were obtained from the Picture Archiving and Communication System (PACS), a comprehensive system providing services with image acquisition, display, storage, transmission and management. Clinicopathologic and immunohistochemical factors were acquired from the Hospital Information System (HIS), an integrated system with powerful clinical decision-support capabilities in a wide variety of clinical areas such as radiology, nurse station and so on. We know that clinical pathological factors such as human epidermal growth factor receptor (her-2), Estrogen Receptor (ER), Progesterone Receptor (PR), and ki-67 are important factors in the diagnosis of ALN metastasis status of breast cancer. Because of our research is a retrospective study, the result of ki67 is not contained in every patient in this study, so this study selected ER and PR as pathological factors. Therefore, baseline clinicopathologic and immunohistochemical data, including age, preoperative histological TNM stage, tumor location, ER status and PR status was acquired from HIS records. The flowchart of this study is shown in Fig. 1. In order to compare the accuracy of the prediction of ALN metastasis in this study with the accuracy of ALN metastasis diagnosed by ultrasound, the ALN status of ultrasound diagnosed also added in the nomogram as a clinical factor (US ALN status). There are 72 ALN negative patients (55 patients in primary cohort and 17 patients in validation cohort) and 75 ALN positive patients (55 patients in primary cohort and 20 patients in validation cohort) diagnosed by ultrasound.

### Mammography acquisition and segmentation

In our study, all patients were undergoing mammography examination within 10 days prior to surgery using Hologic Mammography system (Selenia, US Hologic), which is equipped with molybdenum gold metal ball hall and DR amorphous selenium direct digital plate detector. The acquisition parameters included detector size (24 × 29 cm), matrix (3328 × 4096), limit spatial resolution (7.14 LP/mm), standard pixel size (70 μm), and automatic exposure control. We used mammography Digital Imaging and Communications in Medicine (DICOM) for further feature extraction in the craniocaudual and mediolateral oblique positions^12^.

Mammography images were analyzed by two radiologists (a young radiologist who had 6 years of experience, a senior radiologist who had more than 10 years of experience). Both of the reviewing radiologists were blinded to the pathological results of ALN metastasis. The region of interest (ROI) covered the whole tumor region and was segmented by the 3D Slicer software using the image intensity-based semi-automatic threshold segmentation method^13–16^. The intensities of the tumor area were significantly different from the normal area on the mammography. Therefore, the intensity of the tumorous boundary area was selected as the threshold of the semi-automatic threshold segmentation. ROI was used as the input to extract quantitative radiomics features after segmentation

### Feature extraction

Four groups of radiomics features were extracted from the segmented ROIs of tumors using Matlab R2016b software^17,18^, including first-order, texture, shape and wavelet features^19^. The formula of feature calculation is shown in the Supplementary Data.

### Feature selection, radiomics signature construction and statistical analysis

Radiomics features for each patient were normalized with Min-max normalization method so as to acquire the same distribution of features prior to feature selection. Feature selection was required to reduce over-fitting, redundancy or any other type of bias in our radiomics analysis. All the extracted features and ALN status were used as the input vectors of feature selection and were divided into the independent variable X (radiomics features) and the dependent variable Y (ALN status). The least absolute shrinkage and selection operator (LASSO) regression method was used to select features^20^ and in the meanwhile we carried out bootstrapping method to reduce the estimated bias of feature selection^21^. Then, a radiomics signature was constructed for each patient based on the selected features and their corresponding coefficients obtained by the LASSO regression method.

For the statistical analysis, the correlation between ALN status and radiomics signature, age, T stage, tumor location, US ALN status, ER and PR status was analyzed using SPSS software. Due to non-standard normal distribution of the radiomics features, Spearman correlation analysis was applied. The correlation between each variable and ALN status was observed (p < 0.01, p < 0.05, p > 0.05 indicate significant correlation, general correlation and non-correlation, respectively). In addition, the univariate analysis was used to ascertain a balanceable distribution across all factors of the patients^8^.

### Evaluation of radiomics signature

First, we evaluated the association of the radiomics signature with ALN status in the primary cohort first, and then validated them in the validation cohort. In this study, the support vector machine (SVM) method was used to discriminate the ALN status of each patient in both cohorts^22,23^. The SVM classification method with Gaussian kernel was applied to calculate the area under the receiver operating characteristic curve (AUC) of the radiomics signature. Ten-fold cross-validation was employed to determine the optimal regularization parameter, which is the maximized AUC. After regularization parameter was selected, the AUC of radiomics signature from the validation cohort was calculated using the same method. In addition to AUC, classification accuracy, true positive rate (TPR) and true negative rate (TNR) were also calculated as metrics to assess the quantitative discrimination performance of the radiomics signature in both the primary and validation cohorts^23,24^.

### Development of the radiomics nomogram

First, we conducted a multivariable logistic regression analysis of the clinicopathologic and immunohistochemical factors, including age, T stage in the pathological TNM stage, tumor location in breast quadrant, the status of ER and PR from immunohistochemical results, US ALN status and radiomics signature. We then used the Backward step-wise selection method as the stopping rule via the likelihood ratio test with Akaike’s information criterion^25,26^. Moreover, the multivariable logistic regression analysis was subjected ten-fold cross-validation to achieve a comparatively corrected performance. Based on the results from the multivariable logistic analysis, a radiomics nomogram was constructed^8,27,28^.

### Evaluation of the radiomics nomogram performance

The predictive accuracy and discriminative ability of the radiomics nomogram were determined by the calibration curve^29,30^ and Harrell’s C-index. Calibration curve shown the difference between actual rate of ALN metastasis and predicted probability from radiomics nomogram. Calibration curve is closer to the diagonal dotted line represent a better prediction effect.

C-index refers to the consistency Index, which is generally used to evaluate the predictive power of the model, and the effect in the model can be equal to AUC. C-Index is calculated by (the number of consistency pairs/useful pairs). Consistency pairs refer to the combination that actual observed value are in accordance with the category of the predicted results. Useful pairs refer to the combination that excludes the combination of unreasonable prediction results or actual observation fails to reach the observation point (In the calculation of C-Index, all subjects are required to combine pairs at random). The model was subjected to a ten-fold cross validation to achieve a comparatively corrected performance.

Next, we used the validation cohort to test the performance of the radiomics nomogram. The multivariable logistic regression analysis of the nomogram was applied to the patients in the validation cohort. We compared the performance of the estimated probability of ALN metastasis with the actual outcomes (the ALN metastasis rate) by nomogram calibration curves in both the primary and validation cohorts.

### Decision curve analysis

Decision curve analysis was used to determine the performance and significance of the radiomics nomogram in clinical use by quantifying the net benefits at different threshold probabilities in the validation dataset^8,19^. Assuming that there is a threshold probability p_t_, if the positive probability is greater than p_t_ for clinical treatment, less than p_t_ is to avoid the treatment. And according to the decision theory that p_t_ is great significance for accepting the necessary clinical treatment and avoiding unnecessary clinical operations. Net Benefits = [TPN − FPN * p_t_/(1 − p_t_)]/sample size.

## Results

### Analysis software

We choose the simple randomization method to divide cohort by using the “sample (X, size, replace = FALSE)” function in R software (“X” is the set contain the serial number of all patients; “size” is the number of random sampling). The function show that “size” patients were selected from X as primary cohort and the remaining patients is divided as validation cohort. Since 147 patients were included in this study and we set “size” equal to “0.75 * X”, 110 and 37 patients were random assigned to primary and validation cohort, respectively. MG segmentation was conducted with 3D slicer software(https://www.slicer.org/). Features extraction was used Matlab R2016b software (A data analysis tool software; the MathWorks, Natick, Massachusetts, https://mathworks.com/products/matlab.html). Statistical analysis was analyzed by SPSS software(A platform offers advanced statistical analysis, a vast library of machine learning algorithms, text analysis, open source extensibility, integration with big data and seamless deployment into applications; https://www.ibm.com/analytics/data-science/predictive-analytics/spss-statistical-software). The feature selection method of LASSO was used the “glmnet” package with the “cv.glmnet” of R software(a free software environment for statistical computing and graphics, https://www.r-project.org/). The building of Signature and histogram was done using the “ggplot2”, “gcookbook” package with the method of “ggsave” and “ggplot”. Nomogram plotting, Nomogram evaluation and plotting of calibration curves was used the “rms” package with the method of “nomogram”, “validate”, “calibrate”. The SVM classifier was used “kernvk” package with the method of “kvcv”, classifier evaluation of ROC curve and AUC calculation was used the “pROC” package with the methods of “predict” and “auc”.

### Clinical factors

We assessed the correlation of ALN status with age, T stage, tumor location, ER and PR status in the primary and validation cohorts, as shown in Table 1. In the primary cohort, US ALN status had the significant correlation with ALN status (p = 0.002); T stage and ER status had the generally correlations with ALN status (p = 0.038–0.043); age, tumor location and PR status had not significant correlations with ALN status (p = 0.275–0.310). The correlation was assessed by Spearman’s two-tailed significance test.

**Table 1:** Characteristics of Patients in the Primary and Validation Cohorts.

Factors	Primary Cohort		P(*p < 0.05)	Validation Cohort		P(*p < 0.05)
	LN Metastasis (+)	LN Metastasis (−)		LN Metastasis (+)	LN Metastasis (−)	
Age(mean ± SD)	55.83 ± 11.26	55.93 ± 10.21	0.400	55.68 ± 10.15	52.93 ± 13.99	0.523
T			0.043*			0.021*
T1	25 (40.98)	13 (26.53)		8 (36.36)	4 (26.67)	
T2	32 (52.46)	28 (57.14)		12 (54.55)	7 (46.67)	
T3	4 (6.56)	5 (10.20)		2 (9.09)	2 (13.33)	
T4	0 (0)	3 (6.13)		0 (0)	2 (13.33)	
Location			0.275			0.960
UIQ	14 (22.95)	11 (22.45)		5 (22.73)	3 (20)	
UOQ	33 (54.1)	28 (57.14)		12 (54.55)	9 (60)	
LIQ	5 (8.2)	4 (8.17)		2 (9.08)	1 (6.67)	
LOQ	9 (14.75)	6 (12.24)		3 (13.64)	2 (13.33)	
ER			0.038*			0.043*
+	20 (32.79)	14 (28.57)		7 (31.81)	3 (20)	
−	41 (67.21)	35 (71.43)		15 (68.19)	12 (80)	
PR			0.310			0.26
+	26 (42.62)	24 (48.98)		9 (40.91)	7 (46.67)	
−	35 (57.38)	25 (51.02)		13 (59.09)	8 (53.33)	
US_label			0.002*			0.026*
+	35 (57.38)	14 (28.57)		19 (86.36)	8 (53.33)	
−	26 (42.62)	35 (71.43)		3 (13.64)	7 (46.67)	
RadScore (median (interquartile range))	−0.147 (−0.669 to −0.210)	−0.054 (−0.321 to 0.074)	<0.01*	−0.370 (−0.539 to −0.116)	−0.208 (−0.441 to 0.007)	<0.01*

*NOTE:* P value is calculated from the univariable association analyses between each of the Factors with the LN metastasis status. The factors mainly includes age, T stage, tumor location, ER and PR status of immunohistochemical results. Abbreviations: LN, lymph node; SD, standard deviation; T, T stage; UIQ, upper inner quadrant; UOQ, upper outer quadrant; LIQ, lower inner quadrant; LOQ, lower outer quadrant; ER, estrogen receptor; PR, progesterone receptor. (*P value < 0.05).

### Feature extraction, features selection and radiomics signature construction

As shown in the method, we extracted 22 first-order features, 11 shape features, 28 gray level co-occurrence matrix (GLCM) features from the segmented mammography image and 238 wavelet features were extracted via wavelet decompose in 7 wavelet channels. A total of 299 features were extracted from the mammography images.

**Figure 2:**
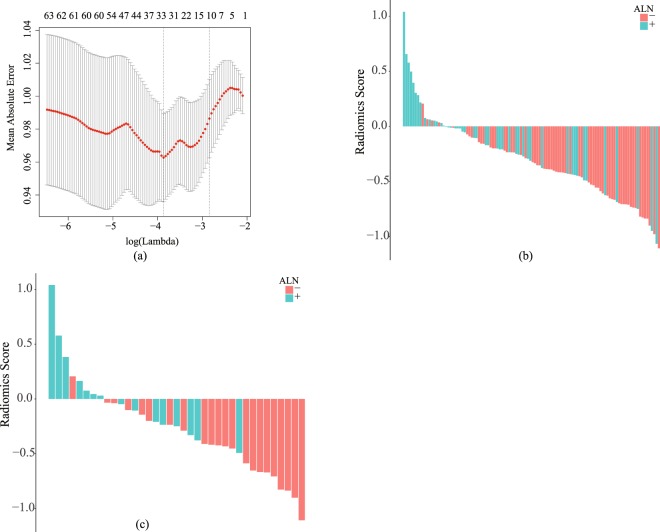
The parameter selection for feature selection is show in (**a**), the radiomics score histogram of primary cohort and validation cohort is shown in (**b**) and (**c**) respectively. The mean absolute error was plotted versus log(*λ*) in (**a**). The positive of ALN metastasis was indicated by red bar, and the negative of ALN metastasis was indicated by blue bar. The y-axis denoted the value of radiomics score in (**b**) and (**c**).

We conducted regularized regression to the extracted features from the primary cohort using LASSO and reduced the feature numbers from 299 to 10 including one non-wavelet feature and nine wavelet features, which were significantly related to ALN metastasis. Figure 2(a) shows the result of the feature selection according to the parameter log(*λ*) and the mean absolute error. In addition, the corresponding coefficients of individual features were also exported from the LASSO analysis.

The selected ten features were used to build the radiomics signature. Radiomics score (Rad-Score) is a manifestation of radiomics signature and contains all the information of the selected features. The Rad-Score was calculated for each patient as a linear fitting of selected features that were weighted by their respective coefficients. The Fig. 2(b,c) show the Rad-Score of the patients in the primary and validation cohorts, respectively. The patients’ ALN metastasis statuses were also indicated with colored bars. The correlation between Rad-Score and ALN status is shown in Table 1.

### The predictive accuracy of the radiomics signature

**Figure 3:**
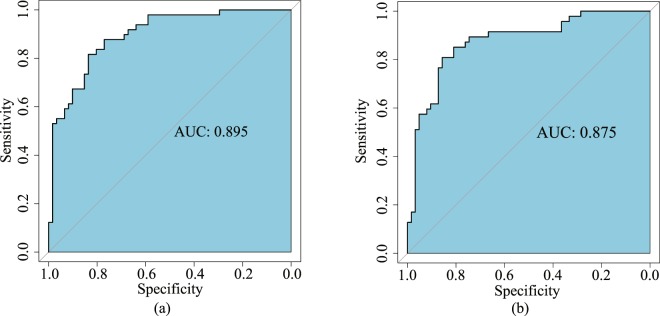
The ROC curves for the primary (**a**) and validation (**b**) cohorts. The AUC for the primary cohort is 0.895 and 0.8725 for the validation cohort.

There was a significant difference in radiomics scores between ALN metastasis and non-ALN metastasis patients in the primary (p < 0.01) and validation (p < 0.01) cohorts. Table 2 shows the performance details of radiomics signature. The AUC and classification accuracy of the radiomics signature from the primary cohort were 0.895 (95% confidence interval [CI], 0.887–0.909) and 84.0% (95% CI, 83.8–84.8%), respectively. The AUC and classification accuracy of radiomics signature from the validation cohort were 0.875 (95% CI, 0.698–0.891) and 80.0% (95% CI, 66.4–83.2%), respectively. The ROC curves of the primary and validation cohorts with AUC values are shown in Fig. 3(a,b). Furthermore, the TPR and TNR of the radiomics signature were 83.6% (95% CI, 82.0–85.2%) and 83.7% (95% CI, 79.5–85.7%) in the primary cohort, 81.8% (95% CI, 72.7–84.9%) and 80.0% (95% CI, 66.7–82.2%) in the validation cohort.

**Table 2:** Performance of the SVM classification model and nomogram.

Index	SVM Classification		Nomogram	
	Primary Cohort	Validation Cohort	Primary Cohort	Validation Cohort
ACC	0.840 [0.838,0.848]	0.800 [0.664,0.832]	0.745 [0.709,0.764]	0.730 [0.702,0.810]
AUC/C-Index	0.894 [0.887,0.909]	0.875 [0.698,0.891]	0.820 [0.752,0.845]	0.809 [0.794,0.833]
TPR	0.836 [0.820,0.852]	0.818 [0.727,0.849]	NA	NA
TNR	0.837 [0.795,0.857]	0.800 [0.667,0.822]	NA	NA

*NOTE:* ACC, accuracy; AUC, area under ROC curve; TPR, True Positive Rate; TNR, True Negative Rate.

### The radiomics nomogram and its performance

**Figure 4:**
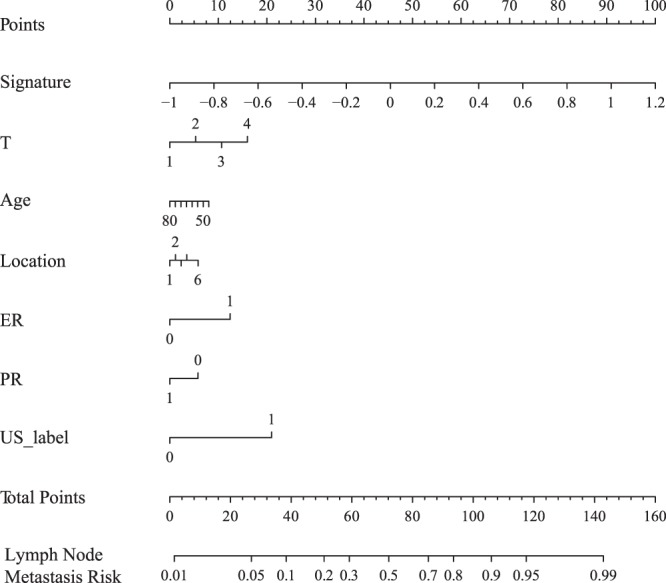
The developed radiomics nomogram by multivariable logistics regression analysis.

**Figure 5:**
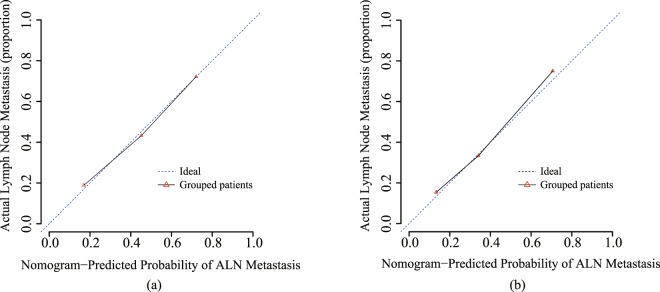
Calibration curves of the radiomics nomograms generated from the primary (**a**) and validation cohorts (**b**). The goodness of fits of predicted probability from radiomics nomograms with the actual outcomes of the ALN metastasis was assessed. The y-axis represents the actual rate of ALN metastasis while the x-axis represents the calculated probability of ALN metastasis. The dashed lines represent the actual diagnosis and the solid line represents the performance of the radiomics nomogram without removed the bias.

We develop a radiomics nomogram for predicting the patients’ ALN status by incorporating radiomics signature with age, T stage, tumor location, US ALN status, ER and PR status using multivariable logistic regression analysis (Fig. 4). The calibration curve was used to estimate the consistent between the radiomics nomogram-predicted probability of ALN metastasis and the actual outcomes. As shown in the Fig. 5(a), the predicted probability of ALN metastasis status is consistent with the actual lymph node metastasis outcomes. The C-index of the radiomics nomogram for the primary cohort was 0.779 (95% CI: 0.752–0.793) and 0.809 (95% CI: 0.794–0.833) for the validation cohort. Good performance was also shown for the probability of ALN metastasis in the validation cohort (Fig. 5(b)).

### Clinical use

**Figure 6:**
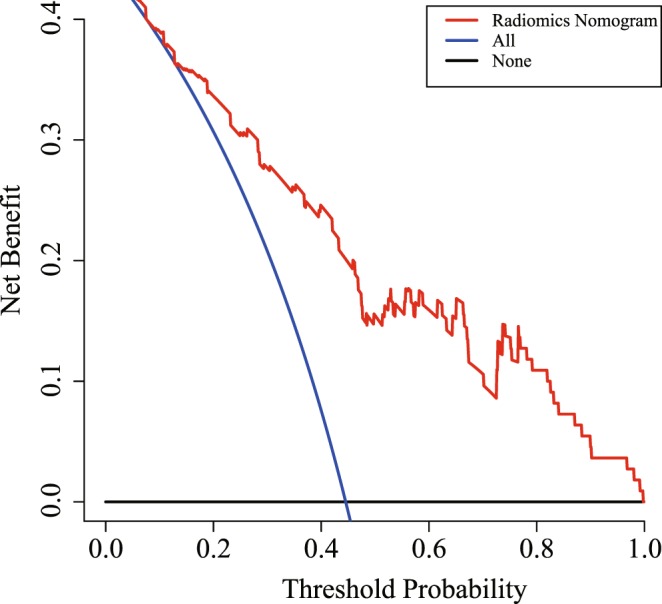
Decision curve analysis for the radiomics nomogram. The x-axis shows the threshold probability and y-axis measures the net benefit. The red line represents the radiomics nomogram. The blue line represents the assumption that all patients showed ALN-positive The black line represents the assumption that no patients showed ALN-positive.

The decision curve evaluated the performance for the radiomics nomogram in terms of clinicopathologic application, thereby, reflecting its clinical usefulness. Within the probability of predicting ALN status ranges of 10% to 100%, more benefit was added from the radiomics nomogram than either the treat-all-patients scheme or the treat-none scheme. Decision curve is shown in Fig. 6. Moreover, although the accuracy of SLNB is higher than this study, the radiomics model have some advantage over SLNB. SLNB is invasive and has some complication. What’s more, sentinel lymph node (SLN) is negative in patients with early breast cancer^31^. This radiomics model is non-invasive and higher sensitivity (83.6% vs 77.1%) and the result of axially lymph nodes metastasis is confirmed by clinical gold standard in this study^32^. In addition to SLNB, there are also several methods in current standard of practice in clinically, including image evaluation, needle aspiration biopsy and pathological biopsy. Ultrasonography and mammography were used for image evaluation in breast cancer before surgery. However, the accuracy of this study is higher than both ultrasonography alone and mammography alone (80.9% vs 71.9% vs 78.4%)^33^. The sensitivity of needle biopsy is lower than this radiomics model (83.6% vs 79.6%)^34^. And this study is non-invasive. Pathological biopsy is the gold standard for clinical diagnosis of axially lymph node metastasis, but the result cannot obtain until postoperative^35^. The process of radiomics model is non-invasive and was completed in preoperative. In short, each step reflects the strengths of our radiomics prediction model.

## Discussion

ALN status is an important factor in developing a personized treatment plan for patients with breast cancer^3^. Currently, SLNB is used as a standard tool to assess the risk of preoperative ALN in clinically node-negative patients with breast cancer. Prior to the American College of Surgeons Oncology Group (ACOSOG) Z0011 trial, patients with 1 or 2 positive SLN via SLNB were considered to be high-risk grade, who would be treated with ALND. However, the ACOSOG Z0011 trial reported that equivalent overall survival for 1 or 2 SLN-positive patients with SLNB alone and SLNB + ALND, both of them all undergoing breast-conserving surgery, whole-breast radiotherapy, and systemic therapy^36–38^. The result supports the notion defining 1 or 2 SLN-positive patients via SLNB as low-risk grade. In addition, the majority of breast cancer patients are SLN negative. There was low incidence of ALN metastases because the size of detected primary tumors has decreased since the public screening mammography programmer was introduced^39^. Furthermore, SLNB is an invasive examination and some studies have also begun to dispute the application of SLNB in the evaluation of preoperative ALN status in patients with breast cancer^40^.

Some related research show that pathological features of primary tumor can predict axillary lymph nodes metastasis in breast cancer^41^. Radiomics features of biomedical images contain information that reflects underlying pathophysiology and these relationships can be revealed via quantitative image analyses^42^. So that quantitative image features of primary tumor can also use to predict axillary lymph nodes metastasis. Second, according to Huang’s and Dong’s study^6,8^, a radiomics signature was concluded by radiomics features that extracted from primary tumor, which used to predict lymph nodes metastasis and show a great performance. Therefore, it is reasonable that we choose the primary tumor as the segmentation region.

There is a great advantage to develop a tool that accurately and non-invasively predict ALN metastasis preoperatively. Establishing prognosis models with clinical factors is a feasible method to evaluate the likelihood of ALN metastasis. For example, Klar *et al*. developed a MSKCC nomogram from 3786 patients undergoing lymph nodes biopsy in a retrospective analysis. This nomogram obtained an AUC of 0.754 in predicting the possibility of lymph node metastasis^43^. In addition, several previous studies have used clinicopathologic, immunohistochemical and genetic factors as independent predictors of ALN metastasis^44^, such as lymphovascular invasion, Ki-67 index, histological grade, molecular subtypes and miRNA. However, the clinicopathologic data are available only after surgery and immunohistochemical examinations. Although genetic analysis had proven to be a reliable method for predicting preoperative ALN status in patients with breast cancer, there are still some limitations such as more expensive and more complex of data acquisition. In clinically, a reliable, accurate, quantifiable and non-invasive methods to predict preoperative ALN status in breast cancer is exactly what doctor need.

Radiomics is a powerful method and has been proved to be able to improve the performance of cancer diagnosis, prediction and decision making^6–8^. In this study, we developed and validated a mammography-based radiomics nomogram for the preoperative prediction of ALN status in patients with breast cancer. For the construction of this radiomics signature, the ROI containing the whole tumor area of the mammography image was segmented and processed, and then a total of 299 features were extracted and reduced to 10 features by the LASSO. Those 10 features that correlated significantly with ALN metastasis were used to construct the signature. The radiomics signature has a favorable discrimination with an AUC of 0.895 in the primary cohort and 0.875 in the validation cohort. In order to allow the clinician to evaluate the risk of ALN metastasis using the radiomics signature, the nomogram was developed by combining the radiomics signature with clinicopathologic and immunohistochemical risk factors, including T stage, age, tumor location, ER and PR status, US ALN status. This nomogram showed an excellent predictive and discrimination power with a C-Index of 0.779 (95% CI, 0.752–0.793) in the primary cohort and 0.809 (95% CI, 0.794–0.833) in the validation cohort. This nomogram provides an accurate and non-invasive tool in predicting the risk of preoperative ALN metastases in patients with breast cancer. Moreover, textural biomarkers objectively reflect the heterogeneity of tumor, and quantifiable features of the radiomics signature have the potential to be ideal true biomarkers^45^. Therefore, this radiomics nomogram may be used as a reliable predictive tool for the ALN status in patients with breast cancer.

At present, there are few studies have predicted lymph node status in breast cancer via radiomics analysis method^7,45,46^. In recently study, Dong and their colleagues used radiomics of T2-weighted fat-suppression and diffusion-weighted MRI to preoperatively predict SLN metastasis with an AUC of 0.863 in primary cohort and 0.805 in the validation cohort in breast cancer. In addition, ultrasound-guided biopsy can play a very important role in preoperative evaluation, but low sensitivity affects its assessment. In the study of Nehmat Houssami^47^ and the study of V. Kuenen-Boumeester^48^, ultrasound-guided lymph node biopsy has the limitation of low sensibility in diagnosis lymph node metastasis. This study is even more prominent than previous studies because the radiomics nomogram that we developed was based on the mammography data. On the one hand, mammography is widely used for breast cancer diagnosis in clinically, which can offer sufficient data to meet the requirement of radiomics analysis. On the other hand, mammography-based radiomics study has a lower cost than other radiomics studies, e.g. MRI-based radiomics. In addition, our mammography-based radiomics nomogram can help clinicians to evaluate the risk of ALN metastasis easily. With this radiomics nomogram tool, the patients with low risk of ALN metastasis can avoid ALND and SLNB. Oppositely, the patients with high risk of ALN metastasis, ALND should be conducted directly and SLNB is no longer necessary.

This study has several limitations. First, a larger number of patients are needed to acquire more reliable evidence for clinical application. Second, the data of our radiomics model established and validated in our study were all from the same hospitals of China, so that still need a multiple center to carry out external validation for the model. Third, studies indicate that genomic factors have a good association with lymph nodal metastasis^46^. Therefore, the integration of genomics signatures may further enhance the ability of radiomics nomograms to predict ALN status in patients with breast cancer in future. On the one hand, the further research of this study is firstly increase the number of patients. On the other hand, we will add an independent validation cohort comprises the patients of several different hospital or different complexion to further validate the performance of this radiomics model. Moreover, due to this study was selected the primary tumor area as region of interest, it was demonstrated that the features of primary tumors also affect ALN metastasis in patient with breast cancer. Therefore, we think that using the tumor microenvironment as region of interest may develop a great performance in clinical research. Finally, when the performance of the radiomics model meets the accreditation criteria: accuracy conform to 94–98.6%, Sensitivity conform to 77.1–93.3%, the clinical application and decision making will start^49^.

In summary, our radiomics nomogram is a reliable and non-invasive predictive tool for preoperative prediction of ALN status and can be conveniently used to optimize current treatment strategy for breast cancer patients.

## Supplementary information

Supplementary
