# Supplementary material for: Preoperative Prediction of Axillary Lymph Node Metastasis in Breast Cancer Using Mammography-Based Radiomics Method
Source: Sci Rep. 2019 Mar 14;9:4429. doi: 10.1038/s41598-019-40831-z (PMC6418289; doi:10.1038/s41598-019-40831-z)
Supplement: Supplementary file 1 — Supplementary [file 41598_2019_40831_MOESM1_ESM.docx]

# Preoperative Prediction of Axillary Lymph Node Metastasis in Breast Cancer Using Mammography-Based Radiomics Method

Jingbo Yang^a,#^, Tao Wang^b,#^, Lifeng Yang^a^, Yubo Wang^a^, Hongmei Li^c^, Xiaobo Zhou^d,*^, Weiling Zhao^d^, Junchan Ren^a^, Xiaoyong Li^c^, Jie Tian^a^, Liyu Huang^a,*^

*^a^ School of Life Science and Technology, Xidian University, Xi’an, Shaanxi 710071, China*

*^b^ Department of Radiology, Shaanxi Provincial People's Hospital, Xi’an, Shaanxi 710068, China*

*^c^ Department of Breast Diseases, Yan’an University Affiliated Hospital, Yan’an, Shaanxi 716000, China*

*^d^Department of Radiology, Wake Forest School of Medicine, Medical Center Boulevard, Winston-Salem, North Carolina 27157*

^#^The authors have contributed equally to this work.

*Corresponding authors.

E-mail addresses: [zhouxb2015@163.com](mailto:zhouxb2015@163.com) (X. Zhou) or huangly@mail.xidian.edu.cn (L. Huang)

**Appendix 1. Inclusion and Exclusion Criteria:**

The inclusion criteria were (1) patients who underwent surgery for breast cancer with curative intent; (2) lymph node biopsy performed; (3) immunohistochemical results included in the report of clinical; (4) standard mammography performed less than 7 days before surgical resection. The exclusion criteria were (1) postoperative complication; (2) patients accepted any therapy preoperative; (3) tumor level grade unknown; (4) has familial hereditary history of cancer.

**Appendix 2. Features and Formulas:**

First-order histogram features consisted of quantified tumor intensity characteristics with first-order statistics calculated from the histogram of all tumor intensities, such as histogram probability estimation, delta, skewness, kurtosis, energy, entropy, max, min, range, median, mean, mean squared error, mean absolute deviation, mean of energy, variance of energy, mean of entropy, variance of entropy, mean of inertia moment, variance of inertia moment, mean of correlation, variance of correlation, root mean square. Two dimensional shape features were based on the quantification of the tumor surface heterogeneity, such as perimeter, area, rectangularity, elongation, circularity and seven invariant matrix features. And two-dimensional features of gray level co-occurrence matrix(GLCM) were extracted to compose the texture features, such as energy, entropy, moment of inertia of an area, correlation, contrast, uniformity, homogeneity, sum of average, variance, non-similarity, autocorrelation coefficient, saliency of region, shadow of cluster, trend of cluster, difference of entropy, sum of entropy, sum of variance, reciprocal of variance, normalized inverse torque, normalized deficit, maximum probability of GLCM. In addition, wavelet transform features were comprised by focusing on the various frequency scales and different feature orientations within the tumor volume. The calculation formula of some features is as following:

1. Skewness describes the asymmetry of histogram distribution in the processed image area:

$$skewness=\frac{1}{\text{σ}^{3}}\sum_{\text{i=0}}^{\text{D-1}} \left( ⅈ-\mu\right)^{3}*\frac{n_{i}}{N},i=0,1\ldots\ldots D-1$$

2. Kurtosis describes the gray value range of the processed image (i.e., ROI region) is around the mean distribution of gray values:

$$kurtosis=\frac{1}{\sigma^{4}}\sum_{i=0}^{L-1} \left( ⅈ-\mu\right)^{4}H\left( ⅈ \right)-3$$

3. Energy describes the uniformity of gray value distribution in the processed image:

$$energy=\sum_{i=0}^{L-1} H\left( ⅈ \right)^{2}$$

4. Entropy describes homogeneity of histogram gray distribution:

$$entropy=\sum_{i=0}^{L-1} H\left( ⅈ \right)\log_{2} \left[ H\left( ⅈ \right) \right]$$

5. Standard deviation describes the arithmetic square root of the mean deviation squared:

$$Standard Deviation=\sqrt{\frac{1}{N}\sum_{i-1}^{N} \left( x_{i}-\mu\right)^{2}}$$

6. Seven invariant matrix describes the seventh order moment characteristic of the invariant moment HU moment is respectively:

$$H1=Z20+Z02$$

$$H2=\left( Z20+Z02 \right)^{2}+4Z{11}^{2}$$

……

1. Rectangularity describes the ratio of the area of an object to the smallest external rectangular rectangle:

$$rectangularity=S/S_{mer}$$

2. Contrast describes local changes in the image:

$$Contrast=\sum_{i=0}^{{quant}_{k}} \sum_{j=0}^{{quant}_{k}} p\left( i,j \right)*\left( i-j \right)^{2}$$

3. Homogeneity describes the measurement of local gray uniformity in the image.

$$Homogeneity=\sum_{i=0}^{{quant}_{k}} \sum_{j=0}^{{quant}_{k}} p\left( i,j \right)*\frac{1}{1+\left( i-j \right)^{2}}$$

4. SumAverage describes the central tendency of distribution:

$$Sumaverage=\sum_{i=2}^{2N} \left[ iP_{x+y}\left( i \right) \right]$$

5. Variance describes the difference between each variable and the population mean:

$$variance=\frac{\sum{(X-\mu)}^{2}}{N}$$

6. Dissimilarity describes the measure is similar to the contrast:

$$Dissimilarity =\sum_{i=0}^{{quant}_{k}} \sum_{j=0}^{{quant}_{k}} p\left( i,j \right)*\left| i-j \right|$$

7. Autocorrelation describes the correlation between each individual expectation of the random error term in the sample:

$$Autocorrelation=\sum_{i=1}^{N} \sum_{j=1}^{N} ijP\left( i,j \right)$$

8. ClusterProminence describes the level of risk assumed by the null hypothesis:

$$ClusterProminence=\sum_{i=1}^{N} \sum_{j=1}^{N} \left[ i+j-m_{x}\left( i \right)-m_{y}\left( j \right) \right]^{4}P\left( i,j \right)$$

**Appendix 3. The least absolute shrinkage and selection operator and radiomics score.**

The least absolute shrinkage and selection operator (LASSO) linear method is a shrinkage estimation method that may be applied for correlative features selection in high dimensional data. A more refined model that compress some coefficients and set some coefficients to zero is obtained by constructing a penalty function. The basic idea of LASSO is to minimize the sum of squared residuals under the constraint that the sum of the absolute values of regression coefficients is less than a constant, which makes it possible to generate some regression coefficients strictly equal to 0. The complexity adjustment of LASSO regression is controlled by the parameter of log(λ) and α, which will affect the performance of punishment. Therefore, we selected the λ value with the lowest cross-validation error to fit the model and obtained the most significant radiomics features that have non-zero coefficients, as the predictive factor. Features with greater contributions to the model are selected.

Radiomics score, a Manifestation of radiomics signature, was contained all the information about the selected features. Radiomics score combined with clinical factors: age and breast T staging to establish a SVM classification model of predictive lymph node metastasis. Similar to it, radiomics score combined with factors: age, T staging, tumor location, ER and PR to construct a radiomics nomogram. Prior to this, we needed to statistically analyze of age, T staging, location, ER, PR and radiomics score with the corresponding statistical analysis method. The standard formula of radiomics score as follows:

$$Radiomics Score=\sum_{i=1}^{n} {Coefficient}_{i}*{Feature}_{i}+Compensation$$

And the actual formula of radiomics score of this study as follows:

$$Radiomics Score=E_{\mathrm{SD}}*\left( 1.469374e-02 \right)+\mathrm{wfirstorder}_{\mathrm{HLH}}*\left( -3.889062e-04 \right)+\mathrm{wfirstorder}_{\mathrm{HLL}}*\left( -4.712440e-04 \right)+ \mathrm{wfirstorder}_{\mathrm{HLL}}*\left( 8.433268e-13 \right)+\mathrm{wfirstorder}_{\mathrm{LHH}}*\left( 1.642636e-03 \right)+\mathrm{wfirstorder}_{\mathrm{LHL}}*\left( 4.116241e-06 \right)+\mathrm{wglcm}_{\mathrm{HHH}}*\left( 8.309277e-02 \right)+\mathrm{wglcm}_{\mathrm{HLH}}*\left( -3.189992e-03 \right)+\mathrm{wglcm}_{\mathrm{HLH}}*\left( -5.112792e-01 \right)+\mathrm{wglcm}_{\mathrm{HLH}}*\left( 5.607619e-01 \right)+1.192951$$

Appendix Table 1. Extraction Features in datasets.

| **First Order** | **Texture** | **Shape** | **Wavelet Transform** |
| --- | --- | --- | --- |
| Energy, Max, Min, Range, Median, Mean, Mean absolute deviation, Root mean square, etc. | Energy, Contrast, Entropy, Homogeneity, Correlation, Variance, SumAverage, etc. | Area, Compactness, Perimeter, etc. | wFirstOrder,  wGLCM, etc. |

*NOTE:* Each type of feature lists some of the features.

Appendix Table 2. Selection Features in primary cohort.

| **Features** | **Coefficient** |
| --- | --- |
| E_SD | 1.469374e-02 |
| Wfirstorder_HLH | -3.889062e-04 |
| Wfirstorder_HLL | -4.712440e-04 |
| Wfirstorder_HLL | 8.433268e-13 |
| Wfirstorder_LHH | 1.642636e-03 |
| Wfirstorder_LHL | 4.116241e-06 |
| Wglcm_HHH | 8.309277e-02 |
| Wglcm_HLH | -3.189992e-03 |
| Wglcm_HLH | -5.112792e-01 |
| Wglcm_HLH | 5.607619e-01 |
| (Intercept) | 1.192951 |

*NOTE:* E_SD, standard deviation of energy; wfirstorder, the wavelet transforms of the first order feature; wglcm, the wavelet transform of the GLCM; H, L, the channel of the wavelet transform; Intercept, compensation coefficient.


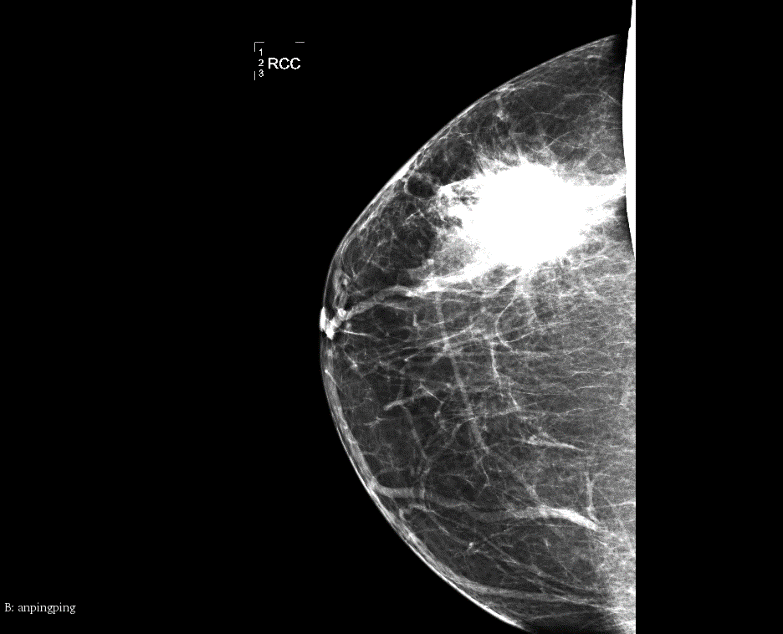
Appendix Figure 1. This figure shows the BC MG image (CC position), the large gray value of the highlight area is the general tumor tissue region in the breast. The glitches around the tumor area are representative of malignant tumors, and the rest are breast tissue, protein, and water. In the image segmentation, only need to divide the tumor area, to retain the tumor area of the original information, the rest of the discard.


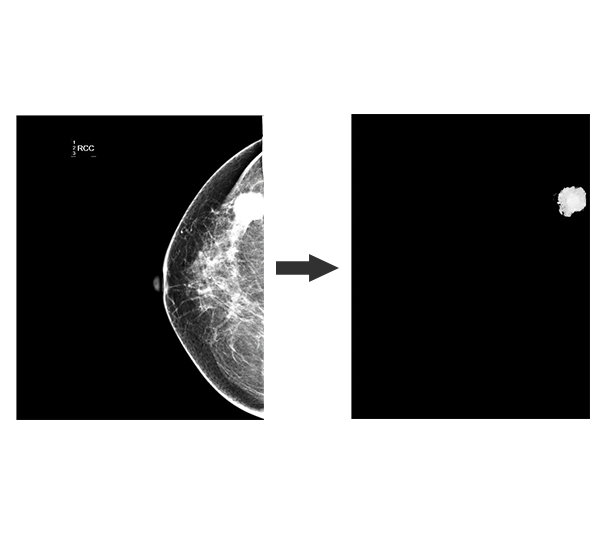


b

a

Appendix Figure 2. The figure of segmentation flow diagram. Figure A shows the original MG before segmentation and figure B shows the region of interest after segmentation (the tumor area).
